# Supplementary material for: Speed-mediated properties of schooling
Source: R Soc Open Sci. 2019 Feb 20;6(2):181482. doi: 10.1098/rsos.181482 (PMC6408369; doi:10.1098/rsos.181482)
Supplement: Supplemental Information [file rsos181482supp1.docx]

**Supplementary Information**

Table S1. Species names, species abbreviations, SL (mean ± s.d.) and the speed and alignment cut-off points between tertiles:

| Species | Abbreviation | SL (cm) | |  | Speed cut-off | |  | Alignment cut-off | |
| --- | --- | --- | --- | --- | --- | --- | --- | --- | --- |
|  |  | mean | s.d. |  | lower | upper |  | lower | upper |
| *M. sp* | BN | 5.92 | 0.83 |  | 3.46 | 4.80 |  | 0.76 | 0.98 |
| *M. sp* | BR | 3.87 | 0.37 |  | 0.93 | 2.35 |  | 0.54 | 0.88 |
| *M. duboulayi* | MD | 4.79 | 0.54 |  | 1.49 | 3.27 |  | 0.61 | 0.91 |
| *M. nigrans* | GC | 5.95 | 0.63 |  | 1.98 | 3.82 |  | 0.64 | 0.96 |
| *M. mccullochii* | SC | 5.31 | 0.59 |  | 1.05 | 2.16 |  | 0.62 | 0.93 |

Effect of speed on linear positioning by species

Figure S1. Speed (BL s^-1^) against proportion of individuals in front or behind the focal individual. BR, BN and GC had a quadratic relationship and are not significantly different. SC and MD had a linear relationship and were not significantly different. Trial averages and s.e. bars are shown.
